# Chronic Bronchitis

**Published:** 1886-11

**Authors:** 


Chronic Bronchitis —

| | | | |
|---|---|---|---|
| ℞ | Tinct. Cubebæ, - - - - | ℥ ss. | |
| | Tr. Benzoin Co., - - - - | ℥ i. | |
| | Tr. Cinch Co., - - - - | ℥ iss. | M. |

Sig.:—Teaspoonful in water every four hours.

